# Nocardia farcinica pneumonia complicated by pneumocystis jiroveci infection in children with Neuromyelitis Optica Spectrum Disorders: a case report and literature review

**DOI:** 10.1186/s13052-024-01827-2

**Published:** 2024-12-02

**Authors:** LingLing Liu, Yuan Huang, SaiNan Shu, Hua Zhou, Feng Fang, Xinglou Liu

**Affiliations:** Department of Pediatrics, Tongji Hospital, Tongji Medical College, Huazhong University of Science and Technology, Wuhan, Hubei Province 430030 China

**Keywords:** Nocardia farcinica, Pneumocystis jiroveci, Neuromyelitis Optica Spectrum disorders, Co-infection, Pneumonia, Case report, Pediatric

## Abstract

**Background:**

Nocardiosis is an opportunistic infection that has a low prevalence rate, its clinical manifestations are atypical and can be easily misdiagnosed as other diseases. The correct diagnosis and treatment are frequently delayed by various factors. In this case report, we present a pediatric patient with Neuromyelitis Optica Spectrum Disorders who developed Nocardia farcinica pneumonia complicated by pneumocystis jiroveci infection.

**Case presentation:**

An 8-year-old girl with chest pain and cough was admitted to the hospital. She suffered from Neuromyelitis Optica Spectrum Disorders and had been taking methylprednisolone and tacrolimus orally for 3 years. She was admitted to the hospital for tests and was diagnosed with acute pneumonia. Despite empiric antibiotic treatment, her condition gradually worsened. Respiratory distress developed, and she needed to use a ventilator for breathing. The symptoms she exhibited led us to suspect the presence of a tumor. Etiological tests later confirmed the co-infection of Nocardia farcinica and Pneumocystis jiroveci. After treatment, the child’s lung infection eventually resolved.

**Conclusion:**

The Nocardia bacteria and Pneumocystis jiroveci are widely distributed in the environment, possess the capability of systemic dissemination, and exhibit significant resistance to specific treatments. Invasive sampling is frequently necessary for confirming their presence. Timely and accurate diagnosis as well as treatment play a crucial role in patient survival.

**Supplementary Information:**

The online version contains supplementary material available at 10.1186/s13052-024-01827-2.

## Background

Nocardia is a rare gram-positive bacteria that is not part of the normal flora of the human body. It can be transported via air, particularly by dust particles, and is widely distributed in soil, decaying vegetables, and aquatic settings worldwide [1]. The majority of infections affect the lungs, which supports the idea that inhalation is the most frequent way for Nocardia to enter the body. Other invasion patterns, however, may be significant in some cases. For example, ingestion of contaminated food can result in disease through gastrointestinal pathways. Skin diseases are usually caused by direct infection with Nocardia bacilli after trauma (including pricking tree thorns or fragments or animal scratches or bites), surgery, vascular catheterization, infection in lesions, or insect bites [2–4].

Nocardiosis can be classified as cutaneous, pulmonary, neurological, or disseminated, depending upon the location and extent of the lesion [1, 5]. Nocardiosis is generally characterized by its ability to spread to almost any organ, especially the central nervous system, and its tendency to recur or progress despite appropriate treatment. The majority of lung infections are primary. However, Nocardia can spread to the lungs from other sites [6]. Signs and symptoms of Nocardia infection are usually nonspecific, making an early diagnosis and course of treatment quite challenging. To improve people’s understanding of the disease, this article thoroughly analyzes clinical manifestations, laboratory test results, imaging studies, BLAF etiological examinations, and related reviews of the literature.

## Case presentation

**Fig. 1:**
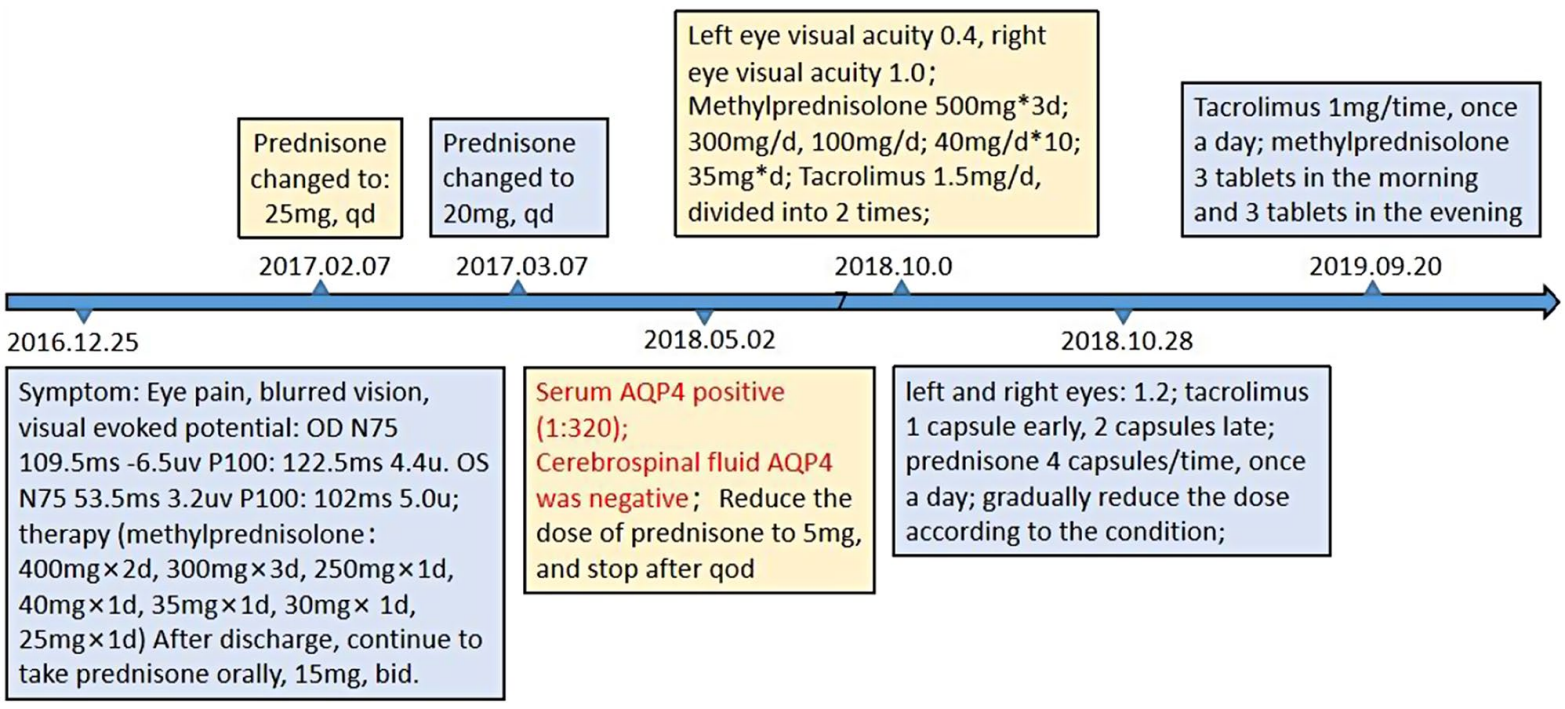
Schematic diagramrepresentation of the diagnosis and treatment of neuromyelitis optica spectrum disorder in children with previousa history of the disorder

**Fig. 2:**
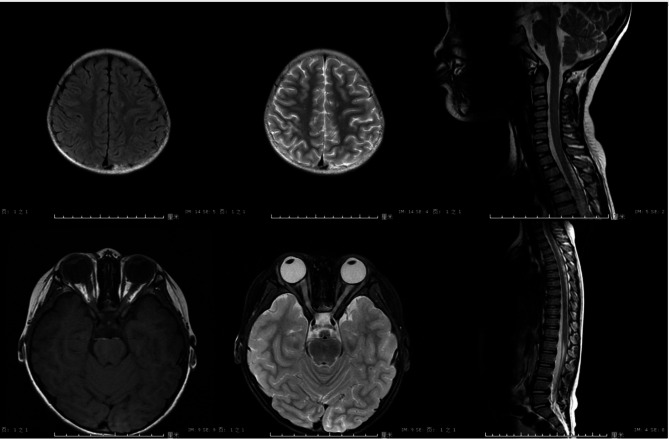
Radiographic changes: MRI showed: Scattered patchy slightly longer T1 and longer T2 signal foci were seen in the left frontoparietal cortex’s subcortical white matter of the left frontoparietal cortex, and, but no obvious abnormal signal foci were found in the remaining brain parenchyma. The bilateral eye rings were intact, no obvious abnormal signal foci were found in the bilateral eyeballs, and no obvious abnormalities were found in the morphological signals of the bilateral optic nerve and optic chiasm. Magnetic morphological signals. There were no obvious abnormalities on magnetic resonance imaging of the lumbar spine and, cervical andspine, or thoracic spine showed no obvious abnormalities

An 8-year-old female patient was admitted to the hospital on December 3, 2019, presenting with left-sided chest pain and cough for a duration of five days. The patient had previously been diagnosed with Neuromyelitis Optica Spectrum Disorders at the same hospital in December 2016 and had undergone regular follow-up appointments at the outpatient clinic. Figure 1 illustrates the diagnosis and treatment of pediatric patients with Neuromyelitis Optica Spectrum Disorders over time, including fluctuations in AQP4 antibody levels. Anti-aqp4 IgG was detected at a titer of 1:320 in May 2018, which decreased to 1:10 in October 2019. In May 2021, Anti-aqp4 IgG was not detected. Magnetic resonance imaging of the head, eyes, lumbar spine, and cervical and thoracic spine is presented in Fig. 2. Methylprednisolone and tacrolimus doses were adjusted according to the patient’s condition. The current treatment regimen includes methylprednisolone (5 tablets daily) and tacrolimus capsules (1 mg in the morning and 0.5 mg in the evening).

Physical examination: A full moon face, palpable enlargement of the superficial lymph nodes, pharyngeal congestion, enlarged tonsils, and thick breath sounds in each lung were discovered during a physical examination.

Laboratory results showed the following blood routine: The patient’s white blood cell count was 14.05 × 10^9^/L(normal range: 3.40–10.80 × 10^9^/L), with a neutrophil percentage of 83.3% (normal range: 29.2–67.1)and a lymphocyte percentage of 12.4%(normal range: 23.6–58.9%). Their red blood cell count was 5.28 × 10^12^/L(normal range: 4.10–5.50 × 10^12^/L), hemoglobin level was at 163.0 g/L(normal range: 120.0–160.0 g/L), platelet count was at 314.0 × 10^9^/L(normal range: 150.0-450.0 × 10^9^/L), C-reactive protein level was elevated at 38.71 mg/L(normal range: 0–10 mg/L), and procalcitonin level was slightly elevated at 0.44 ng/mL (0-0.5 normal; 0.1–0.5 Local infection; ≥2.0 Severe infection). IgA: 0.78 g/L (normal range: 0.11–1.45 g/L), IgG: 7.0 g/L (normal range: 3.3–12.3 g/L), IgM: 0.78 g/L (normal range: 0.33–1.75 g/L), complement C3: 1.58 g/L (normal range: 0.65–1.39 g/L), complement C4: 0.28 g/L (normal range: 0.16–0.38 g/L); Legionella pneumophila type I/Mycoplasma pneumoniae/Rickettsia Q fever/Chlamydia pneumoniae/adenovirus/respiratory tract Syncytial virus/Influenza A virus/Influenza B virus/Parainfluenza virus type 1/2/3- IgM: negative; GM test-Aspergillus galactomannan test: Fungus (1–3)-β-D Dextran: 114.98 pg/mL (normal range: <90pg/ml), Candida mannan: 25.76 pg/mL (<50pg/ml), Aspergillus galactomannan: 0.02 GM index I (normal range: <0.5); Epstein-Barr virus DNA assay < 500.00 copy/mL(normal range:0-500 copy/mL); Tuberculosis infection T cell test (T-SPOT): no reactivity; Mycobacterium tuberculosis smear: no acid-fast bacilli were detected.

Over a period of six days, empiric anti-infectives such as voriconazole, cefoperazone tazobactam, and teicoplanin were administered. During the subsequent re-examination, the child’s C-reactive protein (CRP) levels continued to escalate, reaching 54.08 mg/L, while there was a rapid increase in pulmonary mass. As time progressed, the child exhibited symptoms including dyspnea, exacerbated chest pain, and a poor mental state. By December 11, the child’s blood oxygen saturation had dropped to 88%, and continuous positive airway pressure (CPAP) ventilator was used to assist breathing with the informed consent of family members. When breathing difficulty could not be relieved, tracheal intubation and invasive ventilator support were administered with the guardian’s approval. For neuromyelitis optica spectrum disorders, the child had been on tacrolimus and hormones for a long time. Despite receiving anti-infective treatment, the child’s condition failed to improve and rapidly progressed to dyspnea. Additional tests were necessary to rule out the possibility of tumor diseases. While bacterial and fungal co-infection could have contributed to the rapid progression of lung lesions in children, tumor diseases also remained a potential factor.

Then, subsequently, the child’s tumor-associated biomarkers were evaluated and yielded the following results: elevated levels of carbohydrate antigen 125 at 121.2 U/mL (normal range: 0–35 U/mL), carbohydrate antigen 19 − 9 at 30.02 U/mL (normal range: 0–37 U/mL), and carbohydrate antigen 15 − 3 at 16.3 U/mL (normal range: 0–31 U/mL); normal levels of total β-hCG (< 0.10 mIU/mL) and squamous cell carcinoma-associated antigen 0.4 ng/mL(≤ 1.5ng/mL); slightly elevated alpha-fetoprotein 1.05 ng/mL(≤ 7.0ng/mL) and carcinoembryonic antigen 1.38 ng/mL(≤ 5ng/mL); pleural effusion flow cytometry report showed: the proportion of lymphocytes was not high, the proportion of each subgroup was typically normal, and there was no evident abnormal monoclonal lymphocyte group; no myeloid blasts were found, and the neutrophils were mature granulocytes; pleural effusion test: the color was light yellow, clear Turbidity opacity, Li Fanta test: positive, red blood cell count 400 × 10^6^/L, nucleated cell count 2250 × 10^6^/L, neutrophils 87%, lymphocytes 11%, macrophages 2%, glucose 10.44 mmol/L, total protein 48.4 g/L(normal range:66.0–87.0 g/L), albumin 28.8 g/L(normal range:40.0–55.0 g/L), lactate dehydrogenase 1210 U/L(normal range:125-250U/L),, chyle test was negative. Pleural effusion carcinoembryonic antigen: negative, pleural effusion smear examination showed more lymphocytes, neutrophils, a few mesothelial cells, and macrophages.

Diagnosis and treatment: From December 11 to December 22, the child was subjected to CPAP-assisted ventilation and tracheal intubation in the intensive care unit, with mechanical ventilation administered for one week (December 11 to December 18). The child’s condition exhibited significant improvement.

**Fig. 3:**
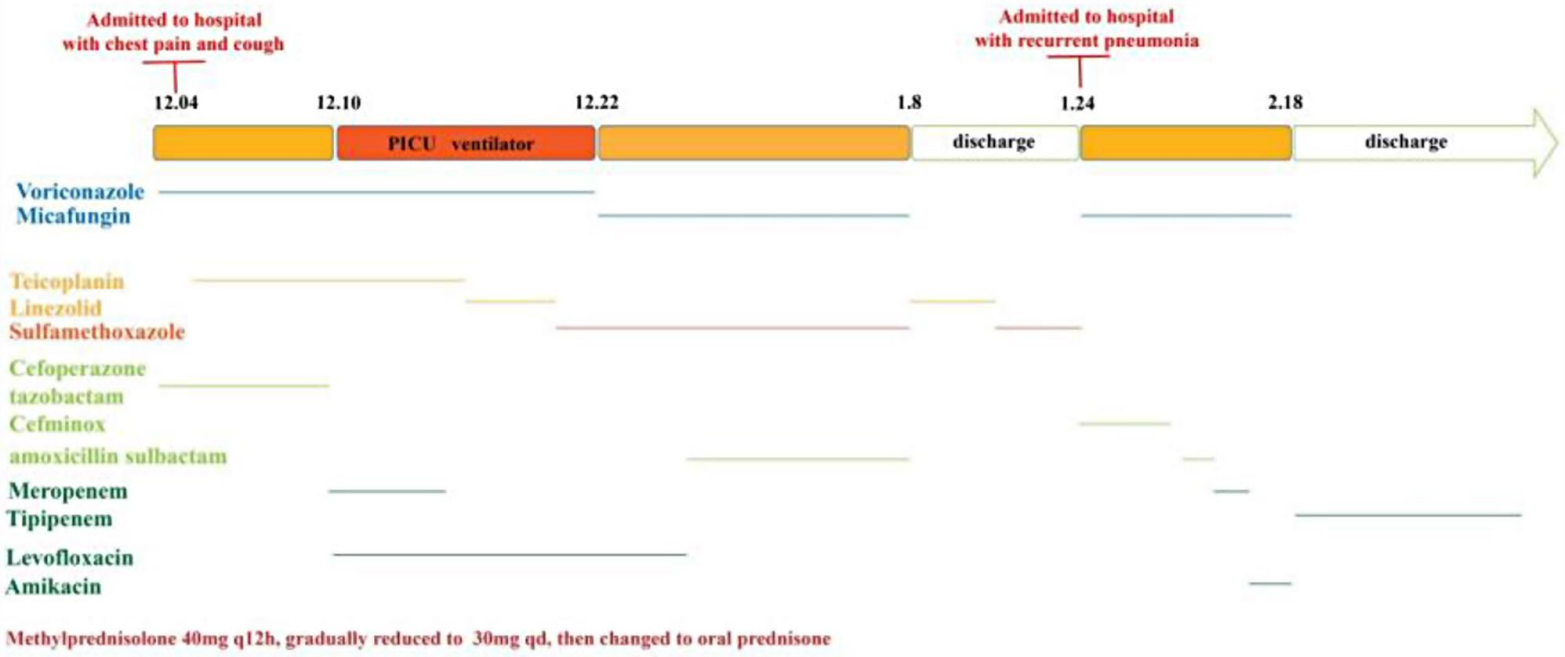
The detailed comprehensive diagnosis and treatment

**Fig. 4:**
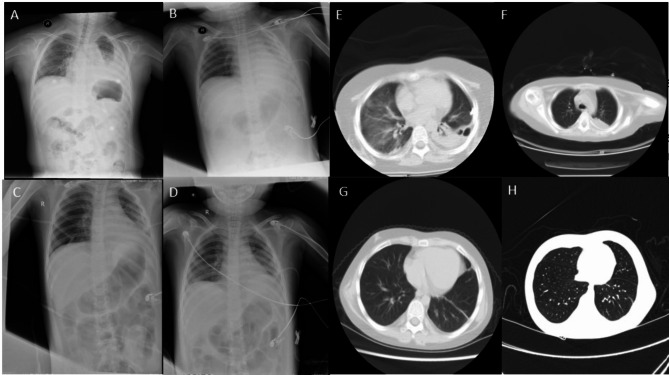
A series of images of lung imaging changes as the child’s condition improved after following treatment. **A**: Left lung infection; left pleural effusion with left lung insufficiency. **B**: Left lung infection, more advancedworse than before. ; The mediastinum has shifted to the left; leftthere is pleural effusion; on the left; a drainage tube wascan be seen in the left pleural cavity; **C**: The infection ofin the right upper lung field is roughly similar to the previous one; the transparency of the left lung is slightly decreasedreduced but slightly higher than the previous one; the. The left pleural effusion is similar to the previous one; before it. **D**: RightThe right upper lung field infection, the left lung infection improved, and the left pleural effusion decreasedall improved. **E**: Left encapsulated fluid-air cavity; bilateral lung infection, insufficiency of bilateral lower lobeslobe insufficiency, and a small amount of pericardial effusion; right pleural thickening. **F**: Left encapsulated fluid-air cavity; bilateral lung infection, insufficiency of bilateral lower lobes, and a small amount of pericardial effusion; right pleural thickening. **G**: SegmentalThe pleural effusion was mostly absorbed, and the left pleura thickened due to segmental insufficiency of the left upper lobe, lingual segment, and left lower lobe, the pleural effusion was basically absorbed, and the left pleura was thickened; **H**: Segmental insufficiency of the left lower lobe, roughly similar to the previous one, with thickening of the left pleura

On the 18th, “sulfamethoxazole” was added to treat the infection, and bronchoalveolar lavage was performed by fiberoptic bronchoscopy. Pleural fluid and Broncho-alveolar lavage samples were separately collected and sent to the BGI-Wuhan Medical Laboratory (BGI-Wuhan, China) for pathogen metagenomic next generation sequencing. mNGS was performed in BGI-Wuhan exactly following the protocol, including DNA extraction, construction of DNA libraries and sequencing. High-quality sequencing data were generated by removing low-quality reads, followed by computational subtraction of human host sequences mapped to the human reference genome (hg19) using Burrows-Wheeler Alignment [7]. The remaining data by removal of low-complexity reads were classified by simultaneously aligning to pathogens metagenomics database (PMDB), consisting of bacteria, fungi, virus and parasites. The classification reference databases were downloaded from NCBI (ftp://ftp.ncbi.nlm.nih.gov/genomes/), encompassing 4,945 whole genome sequence of viral taxa, 6,350 bacteral genomes or scaffolds, 1064 fungi related to human infection, and 234 parasites associated with human diseases. 761 unique sequences were blasted to Nocardia farcinica (Taxonomy ID: 37329) in pleural fluid sample using mNGS data analysis. In a broncho-alveolar lavage sample, 41 unique sequences were blasted to Pneumocystis jirovecii (Taxonomy ID: 42068) and 1469 reads were blasted to Nocardia farcinica (Taxonomy ID: 37329). The patient was infected with Nocardia farcinica and Pneumocystis jiroveci based on the criteria for mNGS positive criteria [8] and clinical symptoms. On January 21, the pleural fluid culture revealed a Nocardia infection which failed to provide susceptibility. After treatment, the levels of CRP and PCT on December 22 were 3.11 mg/L and 0.05 ng/mL, respectively. Figure 3 illustrates the detailed process of diagnosis and treatment, showcasing a series of lung imaging changes that occurred as the child’s condition improved following treatment. Figure 4 portrays the pulmonary examinations.

The child’s family members were very cooperative with the doctor’s treatment and brought her to the hospital on a regular basis for re-examination. Tipipenem was stopped after seven months of continuous oral administration. At the same time, oral immunosuppressive drugs have been used to treat neuromyelitis optica spectrum disorders.

## Discussion and conclusions

Neuromyelitis Optica Spectrum Disorder (NMOSD) is a central nervous system disease. Blindness, paralysis, cognitive impairment, and even death can result from autoimmune inflammatory demyelination disorders. Most patients experience recurrent central nervous system inflammation (CNS inflammation), typically optic neuritis; long-term corticosteroid therapy can increase the risk of hypertension, diabetes, osteoporosis, cushing’s syndrome, and infections, while immunosuppressants increase the risk of infections, including viral, bacterial, and fungal opportunistic infections [9]. Although the precise etiology and pathogenesis of NMOSD remain elusive, it is believed that intricate interplay between genetic and environmental factors contributes to its development. The prevalence of NMOSD has increased overall, likely due to specific environmental factors such as changes in hygiene practices and antibiotic usage. Infectious diseases have been a focus of attention because they are believed to be triggers for many autoimmune diseases and may also contribute to the development or exacerbation of these conditions.10]. Infection may increase the risk of disease recurrence and progression in patients with NMOSD [11]. Scholars believe that tuberculosis bacillus, Helicobacter pylori, intestinal flora, herpes simplex virus, and EB virus may be linked to the prevalence of NMOSD, but more data is needed [10]. It has also been reported that COVID-19 can cause NMOSD relapse [12].

Nocardiosis is an opportunistic infection that usually affects immunocompromised individuals, particularly those with cellular immunodeficiency. Patients with malignant tumors, chronic glucocorticoid users, recipients of organ transplants and hematopoietic stem cell transplants, and HIV-positive individuals are the most frequently infected populations [13]. Normally, Nocardia species are absent in the respiratory tract, and their isolation from sputum samples may indicate infection. The lung is the primary site of Nocardia infection [2, 14]. Nocardiosis typically affects patients with compromised local pulmonary defenses or systemic immunosuppression, and rarely occurs in healthy individuals. Symptoms of Nocardia pneumonia include cough, fever, and breathing difficulties. Because the pathogenesis is `usually subacute or chronic granulomas, Nocardia pneumonia is often confused with other chronic suppurative lung diseases and malignancies, even when there is a high clinical suspicion of the disease. Nocardia can be rapidly diagnosed by examination of sputum, pleural fluid, bronchial lavage fluid, or percutaneous lung aspirate with Gram staining and modified acid-fast staining [15]. It has been reported that respiratory Nocardia isolates may not be pathogenic (i.e., colonizers), but this is extremely rare [14].

Pulmonary nocardiosis may be categorized as acute, subacute, or chronic onset and can not be distinguished by any specific signs or symptoms. However, fever, night sweats, fatigue, anorexia, weight loss, dyspnea, cough, hemoptysis, and pleuritic chest pain have all been reported [16, 17]. In this regard, we summarized the relevant cases of Nocardia pneumonia in children along with their clinical characteristics and treatments in Table 1.

**Table 1:** The relevant cases of Nocardia pneumonia in children along with their clinical characteristics and treatment

NO	Age	Sex	Predisposing factors	Site of Infection	Diagnostic samples	Therapy	Death
1 [18]	11y	male	healthy	lung	Lung abscess drainage fluid culture	Trimethoprim/sulfamethoxazole therapy 6 weeks	no
2 [19]	7y	male	Cystic fibrosis (CF)	lung	sputum culture	Trimethoprim sulfamethoxazole (TMP-SMX), 6 months	no
3 [20]	16y	male	Solid Organ Transplant Recipients: heart re-transplantation	lung, brain	bronchoalveolar lavage fluid and cerebrospinal fluid PCR detection	TMP/SMX, imipenem and moxifloxacin,8 days	yes
4 [21]	17y	male	Cystic fibrosis (CF)/oral corticosteroids for 6 months	lung	sputum specimen	cotrimoxazole (160 mg/800 mg, two tablets b.i.d.) for 9 months.	no
5 [22]	17y	male	acute biphenotypic leukemia received an allogeneic peripheral blood stem cell transplant (PBSCT)	lung	sputum specimen	Meropenem for 2 weeks and MINO for 5 months	no
6 [23]	13y	female	healthy	lung	Cultures of sputum and aspiration material of pulmonary nodules	meropenem and trimethoprim-sulfamethoxazole for 2 months; amoxicillin-clavulanate for 5 months,	no
7 [24]	12y	male	developed chronic ABPA treated with pulse steroids and voriconazole at 6 years	lung	bronchoalveolar lavage fluid	meropenem for 3 months	no
8 [24]	15y	female	CF-related diabetes	lung	Cultures of sputum	TMP/SMX for 7 months	no
9 [24]	13y	male	healthy	lung	Cultures of sputum	TMP/SMX for 4 months	no
10 [25]	17y	male	healthy	lung	biopsy of the lesion	imipenem (500 mg, every 6 h) 2 weeks; oral trimethoprim/ sulfamethoxazole (TMP/SMX) (800 mg/160 mg, every 8 h )for 10 months	no
11 [26]	3y	male	Chronic granulomatous disease (CGD)	lung	submandibular pus; bronchoalveolar lavage fuid (BALF)	meropenem, vancomycin, fuconazole for 3 days; imipenem, linezolid, and voriconazole, sulfamethoxazole for 2 months	yes

All cases we reviewed had pulmonary Nocardia infection, with predisposing factors, including immunosuppression and/or pulmonary disease. Four of the cases had no predisposing factors, and most of the children had underlying diseases or a history of taking hormones, with three having known lung disease. Over a 24-year period, V Pintado et al. [27] summarized the characteristics of Nocardia infection in 34 patients (1978–2001). Immunosuppression and/or lung disease were predisposing factors in 85% of patients; twenty-one patients (62%) had some degree of immunodeficiency due to HIV infection (8 cases), immunosuppressive therapy (8 cases), diabetes (4 cases), or leukemia (1 case). Furthermore, nearly half of the patients (47%) had a prior lung condition, such as chronic obstructive pulmonary disease, silicosis, alveolar proteinosis, or pulmonary fibrosis. Only five patients (15%) did not have a known underlying disease. Attacks are frequently severe and recurring, with significant morbidity and mortality. Ten of the 106 patients died after an average of 4.5 relapses (range 1–10), with four of them succumbing to infection. Three patients died as a result of bronchopneumonia and one as a result of septicemia [28]. Lung Nocardia infections are clinically uncommon, even more so in children with neuromyelitis spectrum disorder, and the majority of these infections are severe. An acute necrotic inflammatory response and nonspecific clinical manifestations characterize Nocardia pulmonary. In our case, the child developed a cough, chest pain, fever, initial dry cough, progressive purulent sputum, and severe dyspnea that worsened despite the use of multiple antibiotics at the outset. Nocardia imaging in the lungs is also nonspecific, and Nocardia infection is difficult and time-consuming to diagnose. Bronchoscopic alveolar lavage with NGS may be the preferred method for rapid pathogen detection [29].

The severity of pulmonary involvement varies from transient infections to fully consolidated bronchopneumonia. Nocardia can cause lung lesions or spread to other organs, particularly in immunocompromised patients following organ or bone marrow transplant or in patients with acquired immunodeficiency syndrome (AIDS). Spread to the bones, joints, skin, kidneys, liver, spleen, and brain can result in serious complications and a high mortality rate. Patients who have received chemotherapy or high-dose corticosteroids are also at risk for developing metastatic infections. Additional risk factors include malignancies (e.g., lymphoma, leukemia) and tuberculosis [16, 30]. A study of 1050 cases of nocardiosis found that systemic infection was responsible for about 32% of the cases. In contrast, pulmonary involvement accounted for 39%, central nervous system involvement for 9%, and skin or lymphatic involvement in 8% of the cases [16]. A single site outside the lung, such as the eye or bone, was involved in 12% of cases. In total, 793 strains of Nocardia were isolated from Observatoire Français des Nocardioses, with the majority coming from the lungs (53.8%). Nocardia farcinica (20.2%), Nocardia abscessus complex (19.9%), and Nocardia nova complex (19.5%) were the most prevalent species. The percentage of N. farcinica increased significantly over time, rising from 13% in 2010 to 27.6% in 2014. Linezolid, amikacin, trimethoprim-sulfamethoxazole, minocycline, and imipenem are the most widely used antimicrobials [31, 32]. The ideal first-line treatment for nocardiosis should include a wide range of species as well as adequate antibiotic concentrations in all involved organs. However, determining the best treatment strategy is difficult because most current antibiotic regimens rely on microbiological data, such as species identification and antimicrobial susceptibility testing (AST) [33–35]. In this case report, the child had nonspecific signs and symptoms and had been taking tacrolimus and hormones for a long time due to Neuromyelitis Optica Spectrum Disorders. The child’s condition developed rapidly and continued to aggravate even with combined anti-infection, leading to difficulty in breathing; as a result, his illness was almost misdiagnosed as a tumor. This is a lesson we should take away from this diagnosis and treatment process.Identifying the causative agent is the most crucial step to rule out infection. Long-term antimicrobial treatment is required for N. asteroids, and possible medications include trimethoprim-sulfamethoxazole, minocycline, amoxicillin-clavulanic acid, cefuroxime, third-generation cephalosporins, amikacin, and imipenem [27]. Excluding patients who died, the average duration of treatment was 6.3 months (range: 3–13 months, median: 6 months). Forty-three patients were administered TMP-SMZ alone (23 patients) or in combination with other antibiotics (20 patients). The most common combination was TMP-SMZ along with imipenem/meropenem (11 patients), of which 5 patients received amikacin at the same time. Linezolid was administered to 3 patients (alone to 2 patients and in combination with meropenem to one patient) Nocardia was suspected as a result of secondary effects on TMP-SMZ (rash and renal tubular acidosis) or in vitro bacteria resistance to TMP-SMZ. Two patients were treated with linezolid alone for 5 and 7 months [36]. Systemic corticosteroid therapy is closely related to pulmonary nocardiosis [37].

Finally, patients with pulmonary nocardiosis frequently have nonspecific clinical symptoms, laboratory tests, and imaging results that are frequently misdiagnosed as other diseases such as actinomycosis, tuberculosis, fungal pneumonia, and even lung tumors and metastases. The risk of nocardiosis must be considered when immunocompromised individuals develop acute, subacute, or chronic pulmonary infections or central nervous system and soft tissue infections. If chest CT shows bilateral consolidation, multiple nodules, or cavity formation, the doctor should consider the possibility of pulmonary Nocardiosis and report any suspicions to the microbiology laboratory to reduce the risk of disease and provide early diagnosis and treatment.

## Electronic supplementary material

Below is the link to the electronic supplementary material.

Supplementary Material 1

## Data Availability

Not applicable.
